# Current Density Imaging Using Directly Measured Harmonic *B*_*z*_ Data in MREIT

**DOI:** 10.1155/2013/381507

**Published:** 2013-03-20

**Authors:** Chunjae Park, Oh In Kwon

**Affiliations:** Department of Mathematics, Konkuk University, Seoul 143-701, Republic of Korea

## Abstract

Magnetic resonance electrical impedance tomography (MREIT) measures magnetic flux density signals through the use of a magnetic resonance imaging (MRI) in order to visualize the internal conductivity and/or current density. Understanding the reconstruction procedure for the internal current density, we directly measure the second derivative of *B*_*z*_ data from the measured *k*-space data, from which we can avoid a tedious phase unwrapping to obtain the phase signal of *B*_*z*_. We determine optimal weighting factors to combine the derivatives of magnetic flux density data, ∇^2^ *B*_*z*_, measured using the multi-echo train. The proposed method reconstructs the internal current density using the relationships between the induced internal current and the measured ∇^2^ *B*_*z*_ data. Results from a phantom experiment demonstrate that the proposed method reduces the scanning time and provides the internal current density, while suppressing the background field inhomogeneity. To implement the real experiment, we use a phantom with a saline solution including a balloon, which excludes other artifacts by any concentration gradient in the phantom.

## 1. Introduction

Magnetic resonance electrical impedance tomography (MREIT) visualizes a cross-sectional conductivity and/or current density inside the human body. The MREIT technique injects currents through attached electrodes in order to probe the imaging subject and then measures the induced magnetic flux density, inside the subject using an MRI scanner. The MRI scanner only measures the *z*-component of the induced magnetic flux density **B** = (*B*_*x*_, *B*_*y*_, *B*_*z*_); therefore the MREIT techniques have focused on the reconstruction of the internal conductivity and/or current density by using the measurable *B*_*z*_ data instead of subject rotation [1–18].

The MREIT techniques used to reconstruct the conductivity and/or the current density have been widely developed and have reached the stage of imaging experiments for live animals and the human body [18, 19]. Due to the poor SNR of measured *B*_*z*_ data in current MREIT experiments, it is critical to reduce the scan time in MREIT, while maintaining the spatial-resolution and sufficient contrast, for practical * in vivo* implementations of MREIT.

In order to increase the quality of measured *B*_*z*_ data, a measurement technique called the injected current nonlinear encoding (ICNE) method was developed, which extends the duration of the injection current until the end of the read-out gradient in order to maximize the signal intensity of the magnetic flux density [20]. Motivated by the ICNE pulse sequence method, an ICNE-multiecho technique was developed and optimized by finding an optimal weighting factor for the multiple measured *B*_*z*_ data [21].

The MREIT technique typically uses an interleaved acquisition, which scans each phase encoding consecutively by injecting two currents possessing positive and negative polarities with the same scan duration and amplitude to double the *B*_*z*_ signal and cancel out the background field inhomogeneity. In order to reduce the scan time, for the measurement of *B*_*z*_, [22] reconstructed the phase signal *B*_*z*_ by filling a partial *k*-space region using the interleaved measurement property.

Functional MRI (fMRI) has been applied to a wide range of neuroscience researches by visualizing neural activities inside the brain in a fast and direct way [23–25]. A fast MREIT imaging technique has been proposed as a promising imaging technique for the continuous monitoring of internal electrical property inside the subject [26]. In this paper, we propose a method to monitor spatial and temporal internal current density changes in the subject by using a fast gradient multi-echo pulse sequence to maximize the measured *B*_*z*_ signal in a short scanning time. Moreover, we derive a direct method to measure ∇^2^ *B*_*z*_ instead of *B*_*z*_ data from the measured *k*-space data. The proposed method can also avoid a tedious unwrapping procedure, which may introduce phase artifact in the recovered phase signal.

For the recovery of the internal current density, we investigate the reconstruction procedure for the internal current density from the measured ∇^2^ *B*_*z*_ data. In the paper [27], a projected current  **J** ^*P*^  was provided by the decomposition **J** = **J** ^*P*^ + **J** ^*N*^, where **J** is the internal current density influenced by the injected current and **J** ^*P*^ is a determined component of **J** from the measured *B*_*z*_ data. The projected current **J** ^*P*^ is identical to the true current **J** when the *z*-component *J*_*z*_ of **J** = (*J*_*x*_, *J*_*y*_, *J*_*z*_) is the same as *J*_*z*_^0^ where *J*_*z*_^0^ is the *z*-component of the background current **J** ^0^.

The projected current **J** ^*P*^ can be determined in a concrete form which consists of the background current **J** ^0^ and the solution of a two-dimensional harmonic equation with the Dirichlet condition that matches the external injection current on the surface of the subject. To recover the internal current density **J** ^*P*^ with the generated *B*_*z*_ caused by the injected current, we only use the second derivatives of *B*_*z*_ and ∇^2^ *B*_*z*_, which are required to solve the two-dimensional harmonic equation for **J** ^*P*^.

To reduce the noise artifact, we apply the ICNE-multi-echo train based on the fast gradient echo and solve an optimal weighting factor of ∇^2^ *B*_*z*_^*ℓ*^,  *ℓ* = 1,…, *N*_*E*_, where *N*_*E*_ denotes the number of echoes at each RF pulse.

In order to verify how the proposed method works, we designed a phantom with a saline solution and fixed a balloon inside the phantom, inflating the balloon by injecting the same saline solution. The phantom was designed to provide a homogeneous magnitude image, but the recovered current density distinguishes inside the balloon. For the experiment, the total scan time was 12.36 seconds to obtain the complete *k*-space data using the interleaved acquisition in order to implement the proposed method with a 128 × 128 spacial matrix size. The phantom experiment demonstrates that the proposed method reduces the scanning time and recovers internal current density, while suppressing the measured noise artifact.

## 2. Methods

 We inject the current *I* through the attached electrodes on a three-dimensional cylindrical conducting object *Ω* with its conductivity distribution *σ*. The injection currents *I* produce the voltage distribution *u* satisfying the following elliptic partial differential equation:

(1) $$\begin{array}{c} \nabla \cdot \left(\sigma \nabla u\right)=0\;\text{in}\;\;\Omega ,\\ -\sigma \nabla u\cdot \nu =g\;\;\mathrm{on}\;\;\partial \Omega ,\;\int_{\partial \Omega }uds=0, \end{array}$$

where *ν* is the outward unit normal vector and *g* is the normal component of the current density on ∂*Ω*. Clearly, ∫_∂*Ω*_ *gds* = 0 due to ∇·(*σ*∇*u*) = 0 in *Ω*. The current density **J** = −*σ*∇*u* and the magnetic flux density **B** = (*B*_*x*_, *B*_*y*_, *B*_*z*_) in *Ω* satisfy the Ampère law **J** = ∇×**B**/*μ*_0_ and Biot-Savart law, where *μ*_0_ = 4*π*10^−7^ Tm/A is the magnetic permeability of the free space. We let *B*_*z*_(*x*, *y*) = *B*_*z*_(*x*, *y*, *z*_0_) where *z*_0_ is the center of a selected imaging slice.

### 2.1. Measurement of *B*_*z*_ Using Interleaved ICNE Acquisition

 For the interleaved ICNE acquisition method, we inject the positive and negative currents, *I* ^+^ and *I* ^−^, through the attached electrodes by scanning each phase encoding consecutively. For a standard spin echo pulse sequence without current injection, the *k*-space MR signal can be expressed as

(2) $$\begin{array}{c} S\left(n,m\right)=\int_{{\mathbb{R}}^{2}}\rho \left(x,y\right)e^{i\delta (x,y)}e^{-i2\pi (\Delta k_{x}nx+\Delta k_{y}my)}dxdy, \end{array}$$

where *ρ*(*x*, *y*) is the real transverse magnetization, *δ* denotes the phase artifact of background field inhomogeneity, and Δ*k*_*x*_ and Δ*k*_*y*_ are the reciprocals of fields of view for the *x* direction and *y* direction, respectively. During the data acquisition, we set

(3) $$\begin{array}{c} \Delta k_{x}=\frac{\gamma }{2\pi }G_{x}\Delta t,\;\;\Delta k_{y}=\frac{\gamma }{2\pi }\Delta G_{y}T_{pe}, \end{array}$$

and sample the data in (2) finitely for

(4) $$\begin{array}{c} n=-\frac{N_{x}}{2},\ldots ,\frac{N_{x}}{2}-1,\;\;\;\;m=-\frac{N_{y}}{2},\ldots ,\frac{N_{y}}{2}-1. \end{array}$$

The names of the above parameters are

 
*γ* = 26.75 × 10^7^ rad/T · s: the gyromagnetic ratio of the proton,
 
*G*_*x*_: the magnetic reading gradient strength,
 Δ*t*: the sampling interval,
 Δ*G*_*y*_: the phase encoding step,
 
*T*_*pe*_: the phase encoding time interval,
 
*N*_*x*_: the number of sampling points,
 
*N*_*y*_: the number of phase encoding lines.

For the conventional MREIT case, we inject the current for the duration of *T*_*c*_^0^ from the end of the 90° RF pulse to the beginning of the reading gradient. In this case, the induced magnetic flux density *B*_*z*_ due to the injection current provides the additional dephasing of spins and consequently the extra phase is accumulated during *T*_*c*_^0^. The corresponding *k*-space data for the injection currents *I* ^±^ can be represented as

(5) SI±(n,m)=∫ℝ2ρ(x,y)eiδ(x,y)e±iγTc0Bz(x,y) ×e−i2π(Δkxnx+Δkymy)dxdy.

Using the notations

(6) $$\begin{array}{c} \rho^{\pm }\left(x,y\right)=\rho \left(x,y\right)e^{i\delta (x,y)}e^{\pm i\gamma T_{c}^{0}B_{z}(x,y)}, \end{array}$$

we can compute the magnetic flux density *B*_*z*_ as

(7) $$\begin{array}{c} B_{z}\left(x,y\right)=\frac{1}{2\gamma T_{c}^{0}}\tan^{-1}\left(\frac{\alpha \left(x,y\right)}{\beta \left(x,y\right)}\right), \end{array}$$

where *α* and *β* are the imaginary and real parts of *ρ* ^+^/*ρ* ^−^, respectively.

In the conventional MREIT case, the noise standard deviation of the measured *B*_*z*_, *sd*_*B*_*z*__, is given as a known quantity, which is inversely proportional to the current injection time *T*_*c*_ and the SNR of the MR magnitude image *Υ* as follows [28, 29]:

(8) $$\begin{array}{c} sd_{B_{z}}\left(r\right)=\frac{1}{2\gamma T_{c}\Upsilon \left(r\right)}. \end{array}$$

Since the ICNE MR pulse sequence injects the current until the end of a read gradient, the total current injection time of the ICNE case is *T*_*c*_^*T*^ : = *T*_*c*_^0^ + *T*_*s*_ and the *k*-space data is represented as

(9) SC±(n,m)=∫ℝ2ρ(x,y)eiδ(x,y)e±iγ(Tc0+Ts/2)Bz(x,y)    ×e−i2π(Δkxnx+Δkymy)dxdy,

where *T*_*s*_ = Δ*tN*_*x*_ is the data acquisition time. In the usual spin echo, the ICNE current injection method demonstrates better SNR in the measured magnetic flux density data than the conventional current injection method. The optimal data acquisition time *T*_s_* has been calculated for the usual spin echo as

(10) $$\begin{array}{c} T_{s}^{*}=\frac{2\sqrt{3}}{3}T_{E}-\sqrt{3}\tau_{rf}, \end{array}$$

which optimally reduces the noise in the *B*_*z*_ data, where *τ*_*rf*_ is the time of RF pulse [30].

In the ICNE MR pulse sequence case, the noise standard deviation of the measured *B*_*z*_,  *sd*_*B*_*z*__^ICNE^, is given as follows [30]:

(11) $$\begin{array}{c} sd_{B_{z}}^{\text{ICNE}}\left(r\right)=\frac{1}{2\gamma \left(T_{c}^{0}+T_{s}^{*}/2\right)\Upsilon \left(r\right)}. \end{array}$$

The prolonged data acquisition time, however, may suffer from undesirable side artifacts such as blurring, chemical shift, and motion artifacts along the phase encoding direction. To reduce the undesirable side artifacts, we divide the prolonged data acquisition time into several short ones in the ICNE-multi-echo MR pulse sequence. 

### 2.2. Measurement of ${\tilde{\nabla }}^{2}B_{z}$ Using ICNE-Multiecho Train

Using the ICNE-multi-echo MR pulse sequence, the measured *k*-space data can be represented as

(12) Sℓ±(n,m)=∫ℝ2ρℓ(x,y)eiδℓ(x,y)e±iγTcℓBz(x,y)  ×e−i2π(Δkxnx+Δkymy)dxdy, ℓ=1,…,NE,

where *N*_*E*_ is the echo number, *T*_*c*_^*ℓ*^ is the *ℓ*th time width of the injected current, and *ρ* ^*ℓ*^ and *δ* ^*ℓ*^ denote the *ℓ*th transverse magnetization and phase artifact, respectively.

**Figure 1:**
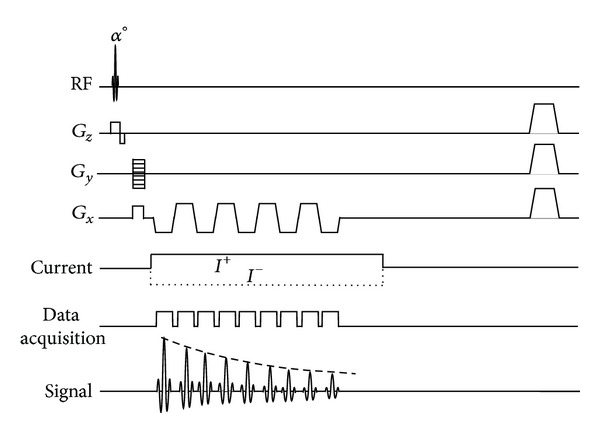
Diagram of the ICNE-multi-echo MR pulse sequence based on a gradient echo.

Figure 1 presents a schematic diagram for the ICNE-multi-echo MR pulse sequence based on a gradient echo pulse sequence. By taking the inverse fast Fourier transform, the ICNE-multi-echo sequence generates multiple complex images with different magnitude amplitudes depending on *T*_2_* decay and different widths of current injection time:

(13) $$\begin{array}{c} \psi^{\ell \pm }\left(x,y\right):=\rho^{\ell }\left(x,y\right)e^{i\delta^{\ell }(x,y)}e^{\pm i\gamma T_{c}^{\ell }B_{z}(x,y)},\;\ell =1,\ldots ,N_{E}. \end{array}$$

Using the relation (13), we derive a formula for ${\tilde{\nabla }}^{2}B_{z}^{\ell }$ as

(14)   ∇~2Bzℓ(x,y)=1iγTcℓ∇~·(|ψℓ+(x,y)|ψℓ+(x,y)∇~ψℓ+(x,y)|ψℓ+(x,y)|  − |ψℓ−(x,y)|ψℓ−(x,y)∇~ψℓ−(x,y)|ψℓ−(x,y)|),

where $\tilde{\nabla }B_{z}^{\ell }=\left(\partial B_{z}^{\ell }/\partial x,\partial B_{z}^{\ell }/\partial y\right)$ denotes the two-dimensional gradient of *B*_*z*_^*ℓ*^. The induced ${\tilde{\nabla }}^{2}B_{z}^{\ell }$ in (14) removes the low-frequency phase artifact *δ* ^*ℓ*^ by subtracting ${\tilde{\nabla }}^{2}\delta^{\ell }$ in (14).

The calculated vector $\left(\left|\psi^{\ell \pm }\right|/\psi^{\ell \pm }\right)\tilde{\nabla }\left(\psi^{\ell \pm }/\left|\psi^{\ell \pm }\right|\right)$ corresponding to $i\gamma T_{c}^{\ell }\tilde{\nabla }\left(\delta^{\ell }\pm B_{z}^{\ell }\right)$ includes unavoidable measured noise. When we consider the decomposed form of $\left(\left|\psi^{\ell }\right|/\psi^{\ell }\right)\tilde{\nabla }\left(\psi^{\ell }/\left|\psi^{\ell }\right|\right)=\;\tilde{\nabla }f+\tilde{\nabla }\times \;\Psi$, where the curl term $\tilde{\nabla }\times \;\Psi$ is a part of unavoidable measured noise, the divergence procedure for ${\tilde{\nabla }}^{2}B_{z}^{\ell }$ in (14) cancels $\tilde{\nabla }\times \;\Psi$, and therefore the measured ${\tilde{\nabla }}^{2}B_{z}^{\ell }$ includes a denoising procedure by suppressing a part of the measured noise.

### 2.3. Optimal Combination of Measured ${\tilde{\nabla }}^{2}B_{z}^{\ell }$,  *ℓ* = 1,…, *N*_*E*_

The measured ${\tilde{\nabla }}^{2}B_{z}^{\ell }$,  *ℓ* = 1,…, *N*_*E*_ includes different amounts of unavoidable noise since the intensity of transverse magnetization and the width of injected current are different at each echo.

 The noise standard deviation of ${\tilde{\nabla }}^{2}B_{z}^{\ell }$ in (14) is given as

(15) $$\begin{array}{c} sd_{{\tilde{\nabla }}^{2}B_{z}^{\ell }}\left(x,y\right)=\frac{C}{\gamma T_{c}^{\ell }\Upsilon^{\ell }\left(x,y\right)}, \end{array}$$

where the constant *C* only relates to the numerical differentiations for ${\tilde{\nabla }}^{2}B_{z}^{\ell }$, and *Υ* ^*ℓ*^ denotes the SNR of the *ℓ*th MR magnitude image.

Since the noise levels of the measured ${\tilde{\nabla }}^{2}B_{z}^{\ell }$,  *ℓ* = 1,…, *N*_*E*_ in (14) are given as known quantities, we can utilize the known information $sd_{{\tilde{\nabla }}^{2}B_{z}^{\ell }}$ to determine an optimized ${\tilde{\nabla }}^{2}B_{z}$ which combines the multiple ${\tilde{\nabla }}^{2}B_{z}^{\ell }$:

(16) $$\begin{array}{c} {\tilde{\nabla }}^{2}B_{z}\left(x,y\right)=\sum_{\ell =1}^{N_{E}}\omega^{\ell }\left(x,y\right){\tilde{\nabla }}^{2}B_{z}^{\ell }\left(x,y\right). \end{array}$$

The problem of determining the weighting factors *ω* ^*ℓ*^ for ${\tilde{\nabla }}^{2}B_{z}^{\ell }$ can be formulated as

(17) min⁡ωℓ(x,y), ℓ=1,…,NE ∑ℓ=1NE(ωℓ(x,y))2Var⁡∇~2Bzℓ(x,y)subject  to ∑ℓ=1NEωℓ(x,y)=1, ωℓ(x,y)>0,

where $\text{Va}{\text{r}}_{{\tilde{\nabla }}^{2}B_{z}^{\ell }}$ denotes the noise variance of ${\tilde{\nabla }}^{2}B_{z}^{\ell }$,  *ℓ* = 1,…, *N*_*E*_ in (14).

Following similar arguments in [21], the weighting factors *ω* ^*ℓ*^ can be determined as

(18) $$\begin{array}{c} \omega^{\ell }\left(x,y\right)=\frac{{\left(T_{c}^{\ell }\right)}^{2}{\left|\psi^{\ell \pm }\left(x,y\right)\right|}^{2}}{\sum_{m=1}^{N_{E}}{\left(T_{c}^{m}\right)}^{2}{\left|\psi^{m\pm }\left(x,y\right)\right|}^{2}},\;\ell =1,\ldots ,N_{E}, \end{array}$$

where *ψ* ^*ℓ*±^ in (13) is the inverse fast Fourier transform of the measured *k*-space data *S* ^*ℓ*±^.

### 2.4. Recovery of Internal Current Density Using the Optimized ${\tilde{\nabla }}^{2}B_{z}$

The internal current density **J** = −*σ*∇*u* and the magnetic flux density **B** = (*B*_*x*_, *B*_*y*_, *B*_*z*_) in *Ω* satisfy the Ampère law **J** = ∇×**B**/*μ*_0_ where *μ*_0_ is the magnetic permeability of the free space. The magnetic resonance current density imaging (MRCDI) technique, which allows the rotation of the object in the MRI scanner, directly visualizes the internal current density by measuring the full components of **B** [31].

The MREIT techniques focus on visualizing the internal current density using only *B*_*z*_ component of **B** without rotating the subject. A cylindrical imaging domain *Ω* can be represented as

(19) $$\begin{array}{c} \Omega =\underset{t\in (-H,H)}{⋃}\Omega_{t},\;\text{where}\;\;\Omega_{t}=\Omega \cap \left\{\left(x,y,z\right)\in {\mathbb{R}}^{3}\;|\;z=t\right\}, \end{array}$$

where *Ω*_0_ denotes the middle slice of the imaging subject *Ω*.

In the paper [27], the only recoverable current from the measured *B*_*z*_ data can be represented as **J** ^*P*^ = **J** ^0^ + J*, where **J** ^0^ = ∇*α* and J* = (∂*β*/∂*y*, −∂*β*/∂*x*, 0). Here, *α* is a homogeneous voltage potential satisfying

(20) ∇2α=0 in  Ω,∇α·ν=J·ν on⁡  ∂Ω, ∫∂Ωαds=0,

and *β*_*t*_(*x*, *y*): = *β*(*x*, *y*, *t*) satisfies the following two-dimensional Laplace equation for each slice *Ω*_*t*_ ⊂ *Ω*:

(21) $$\begin{array}{c} {\tilde{\nabla }}^{2}\beta_{t}=\frac{1}{\mu_{0}}\nabla^{2}B_{z}\;\text{in}\;\Omega_{t},\\ \beta_{t}=0\;\mathrm{on}\;\partial \Omega_{t}, \end{array}$$

where ∇ = (∂/∂*x*, ∂/∂*y*, ∂/∂*z*) and $\tilde{\nabla }\;=(\partial /\partial x,\partial /\partial y)$. From the optimized ${\tilde{\nabla }}^{2}B_{z}$ in (16) on each imaging slice *Ω*_*t*_, we can estimate ∇^2^ *B*_*z*_ in (21).

Equations (20) and (21) show that we can reconstruct the projected current **J** ^*P*^ from the optimized ${\tilde{\nabla }}^{2}B_{z}$ immediately, instead of *B*_*z*_, by solving two-dimensional Laplace equations in the region of interest (ROI). The projected current **J** ^*P*^ provides an optimal approximation of the true current **J** and, moreover, the gap **J** − **J** ^*P*^ depends only on the longitudinal component *J*_*z*_ − *J*_*z*_^0^ of  **J** − **J** ^0^.

### 2.5. Experimental Setup

**Figure 2:**
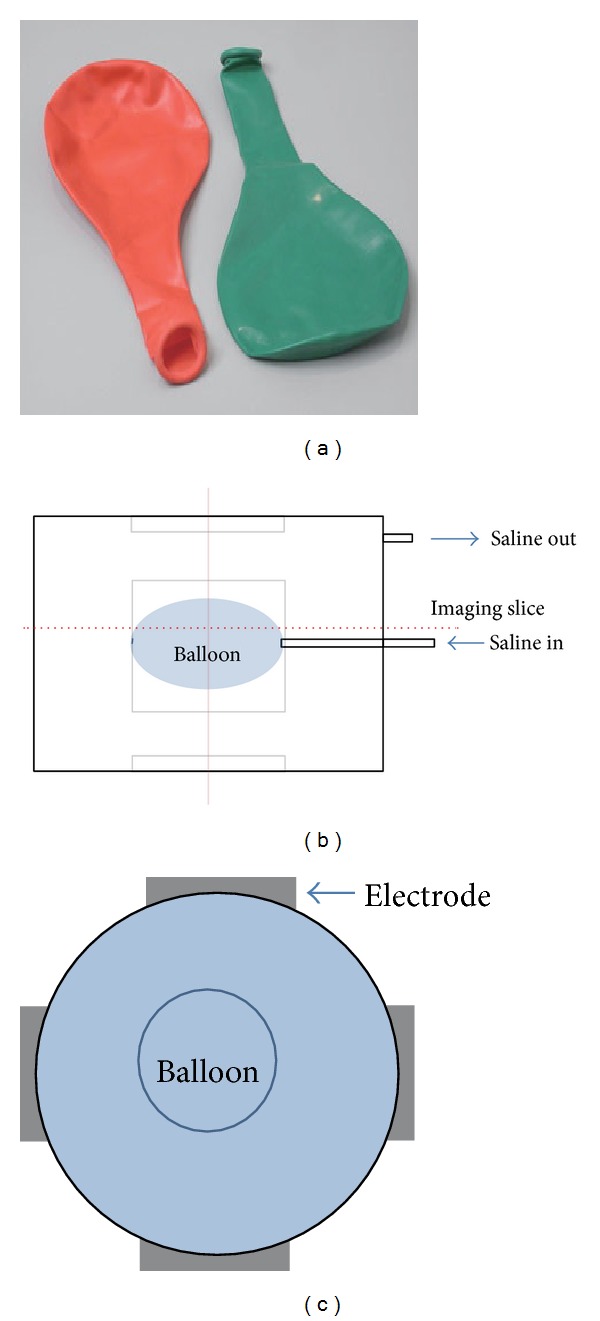
(a) Balloon used for the experiment, (b) and (c) balloon phantom design and the electrodes position, respectively.

 In order to demonstrate the proposed method, we performed a phantom with a saline solution including a balloon for the visualization of internal current density. The internal of the balloon was filled with the same saline solution and the volume of the balloon was controlled by injecting the saline solution, which excluded other artifacts by any concentration gradient in the phantom.  Figure 2(a) illustrates the used balloon for the phantom experiment, and Figures 2(b) and 2(c) show a phantom design to describe how to setup the balloon phantom. 

After positioning the phantom inside a 3.0T MRI scanner (Achieva, Philips), we collected *k*-space data with 8-channel RF coil using the gradient multi-echo ICNE pulse sequence, which extends throughout the duration of the injection current until the end of a readout gradient [20]. The maximum amplitude of the injection current was 5 mA and the total imaging time was 12.36 seconds to measure the interleaved *k*-space *S* ^*ℓ*±^ data, *ℓ* = 1,…, *N*_*E*_. The slice thickness was 5 mm, the number of axial slices was one, the repetition time *T*_*R*_ = 60 ms, the echo spacing Δ*T*_*E*_ = 6 ms, the flip angle was 40 degree, and the multi-echo time *T*_*E*_*ℓ*__ = 6 + (*ℓ* − 1) × 6 ms for *N*_*E*_ = 9. The FOV was 160 × 160 mm^2^ with a matrix size of 128 × 128. The current injection time *T*_*c*_*ℓ*__ for each echo was almost the same as the multi-echo time *T*_*E*_*ℓ*__ = 6 + (*ℓ* − 1) × 6,  *ℓ* = 1,…, 9 because the current was continuously injected until the end of the readout gradient.

## 3. Results

**Figure 3:**
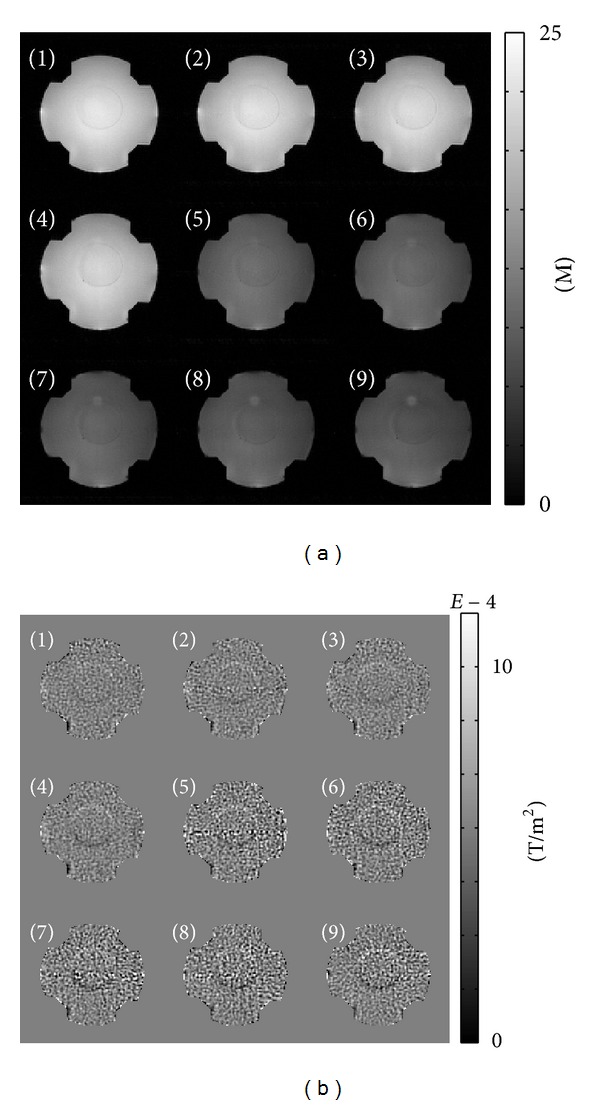
(a) Acquired magnitude images |*ρ* ^*ℓ*^|,  *ℓ* = 1,…, 9, where *ρ* ^*ℓ*^ was the *ℓ*th measured *T*_2_* weighted complex image, (b) measured ${\tilde{\nabla }}^{2}B_{z}^{\ell }$ images using (14) corresponding to the *ℓ*th *k*-space data *S* ^*ℓ*±^,  *ℓ* = 1,…, 9.

Figure 3(a) shows the acquired magnitude images |*ρ* ^*ℓ*^|,  *ℓ* = 1,…, 9, where *ρ* ^*ℓ*^ was the *ℓ*th measured *T*_2_* weighted complex image.  Figure 3(b) shows the measured ${\tilde{\nabla }}^{2}B_{z}^{\ell }$ images using (14) corresponding to the *ℓ*th *k*-space data *S* ^*ℓ*±^,  *ℓ* = 1,…, 9. Inside and outside of the balloon, the MR magnitude images are almost the same because of the same saline solution, but the measured ${\tilde{\nabla }}^{2}B_{z}^{\ell }$ images show distinguishable signals reflecting the conductivity changes inside and outside of the balloon.

Since both sides, inside and outside of the balloon, are homogeneous, the ${\tilde{\nabla }}^{2}B_{z}^{\ell }\approx -\mu_{0}\nabla u\times \nabla \sigma$ should be near zero except the boundary of the balloon without noise effect because the conductivity value is constant in each region. To evaluate the noise level of *B*_*z*_^*ℓ*^, we calculated the discrete *L* ^2^-norm:

(22) Err2∶=||∇~2Bzℓ  ||L2(Ω∖∂D)    =∑(xi,yj)∈Ω∖∂D∇~2Bzℓ(xi,yj)2|Ωij|,

where *Ω* is the imaging ROI region, ∂*D* denotes the boundary of balloon, and |*Ω*_*ij*_| is the pixel size.

Table 1 shows the *L* ^2^-norm, Err_2_, in which the values depends on the *T*_2_* decay rate and the width of injected current.

 The estimated noise levels were reduced up to the 4th echo, but increased in the following echoes because the intensity of magnitude images follows the exponential *T*_2_* decay, and the width of the injected current linearly increases.

**Figure 4:**
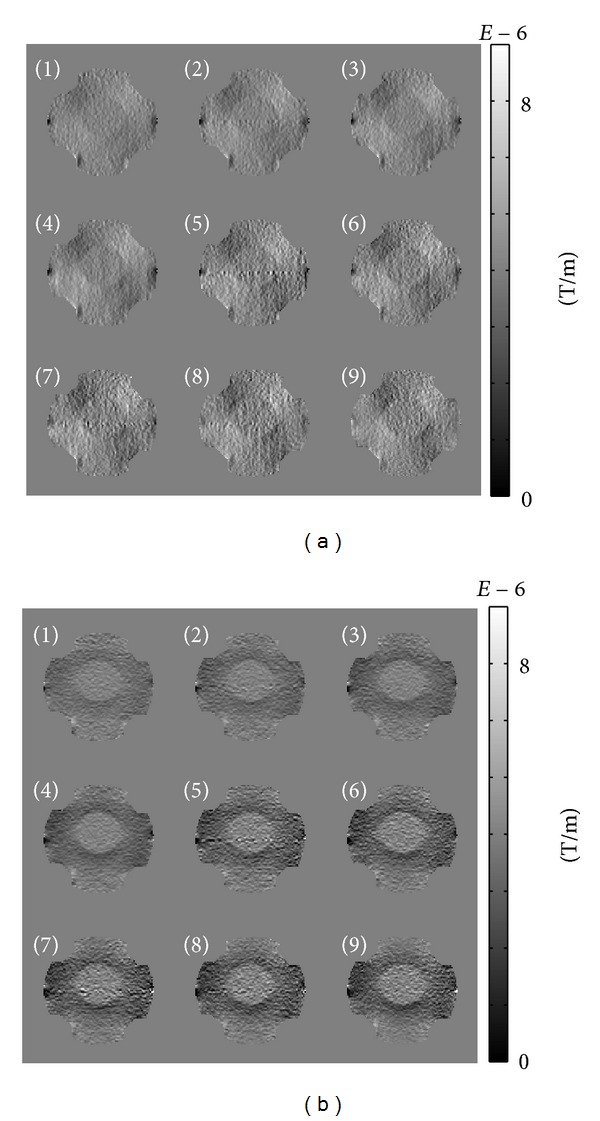
(a) Measured ∂*B*_*z*_^*ℓ*^/∂*x* images, (b) measured ∂*B*_*z*_^*ℓ*^/∂*y* images, *ℓ* = 1,…, 9.

Figures 4(a) and 4(b) show the measured ∂*B*_*z*_^*ℓ*^/∂*x* and ∂*B*_*z*_^*ℓ*^/∂*y* images, respectively, where

(23) ∇~Bzℓ(x,y)=|ψℓ+(x,y)|ψℓ+(x,y)∇~ψℓ+(x,y)|ψℓ+(x,y)|  −|ψℓ−(x,y)|ψℓ−(x,y)∇~ψℓ−(x,y)|ψℓ−(x,y)|, ℓ=1,…,9.

Since the currents were transversally injected, the measured ∂*B*_*z*_^*ℓ*^/∂*y* reflected dominant internal current flows.

**Figure 5:**
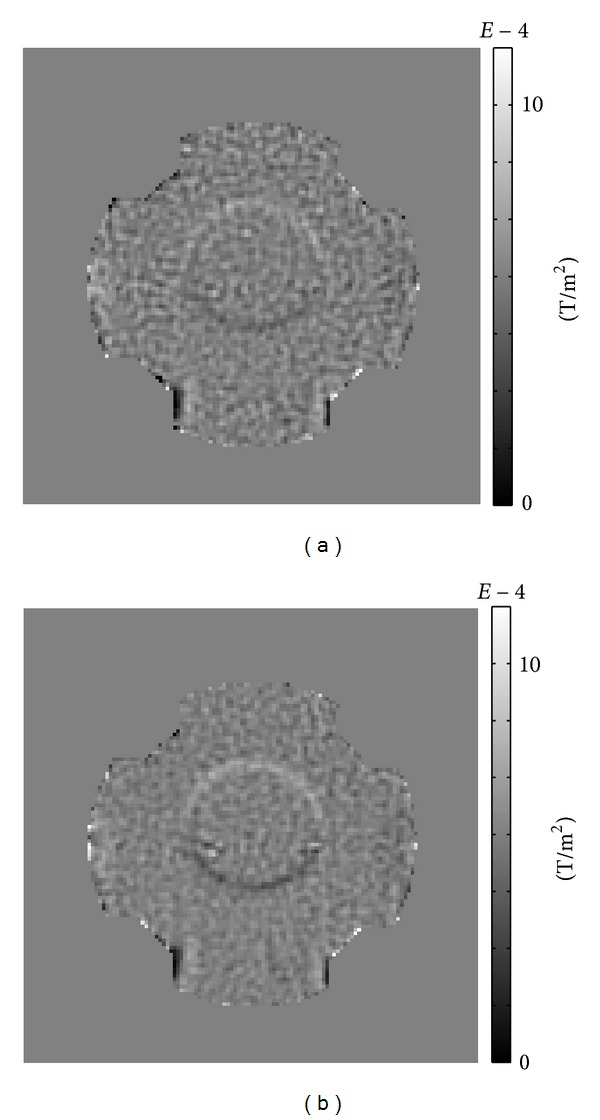
(a) Reconstructed ${\tilde{\nabla }}^{2}B_{z}^{\text{avrg}}=\left(1/N_{E}\right)\sum_{\ell =1}^{N_{E}}{\tilde{\nabla }}^{2}B_{z}^{\ell }$ images, (b) reconstructed ${\tilde{\nabla }}^{2}B_{z}^{\text{opt}}=\sum_{\ell =1}^{N_{E}}\omega^{\ell }{\tilde{\nabla }}^{2}B_{z}^{\ell }$ images, where *ω* ^*ℓ*^ is the weighting factor by solving (17).

Figures 5(a) and 5(b) display the reconstructed ${\tilde{\nabla }}^{2}B_{z}^{\text{avrg}}=\left(1/N_{E}\right)\sum_{\ell =1}^{N_{E}}{\tilde{\nabla }}^{2}B_{z}^{\ell }$ and ${\tilde{\nabla }}^{2}B_{z}^{\text{opt}}=\sum_{\ell =1}^{N_{E}}\omega^{\ell }{\tilde{\nabla }}^{2}B_{z}^{\ell }$ images, respectively, where *ω* ^*ℓ*^ is the weighting factor by solving (17).

**Figure 6:**
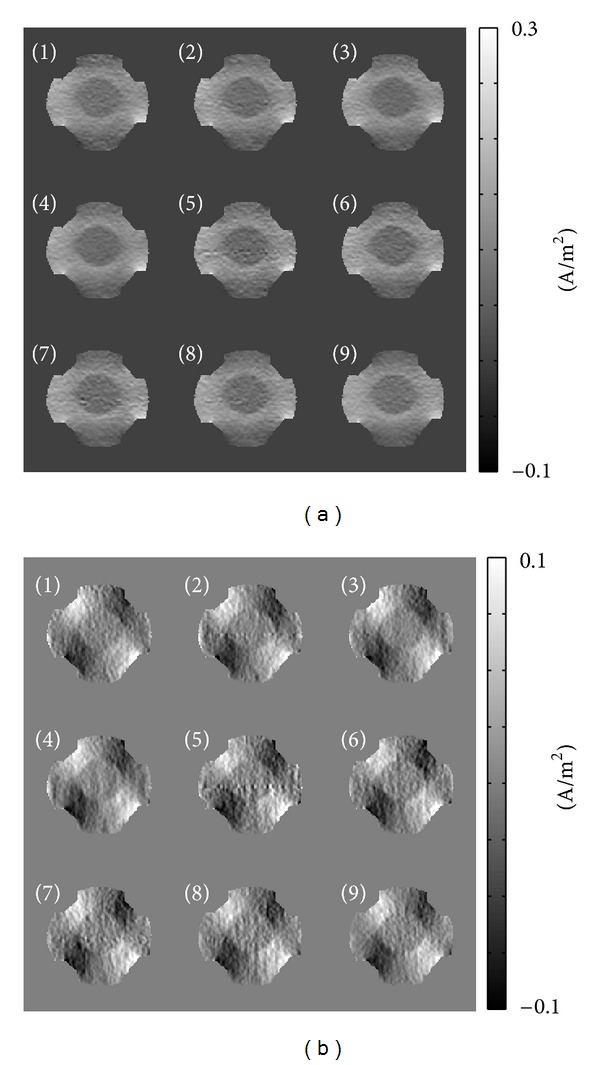
Recovered current density by solving (20) and (21). (a) Recovered *J*_*x*_^*ℓ*^ images, (b) recovered *J*_*y*_^*ℓ*^ images, *ℓ* = 1,…, 9.

Figure 6 shows the recovered current density images, *J*_*x*_^*ℓ*^ and *J*_*y*_^*ℓ*^, corresponding to the *ℓ*th echo. To obtain the current density images, we solved (20) for the background homogeneous current and the two-dimensional harmonic equation (21) to reflect the measured ${\tilde{\nabla }}^{2}B_{z}^{\ell }$ data.

**Figure 7:**
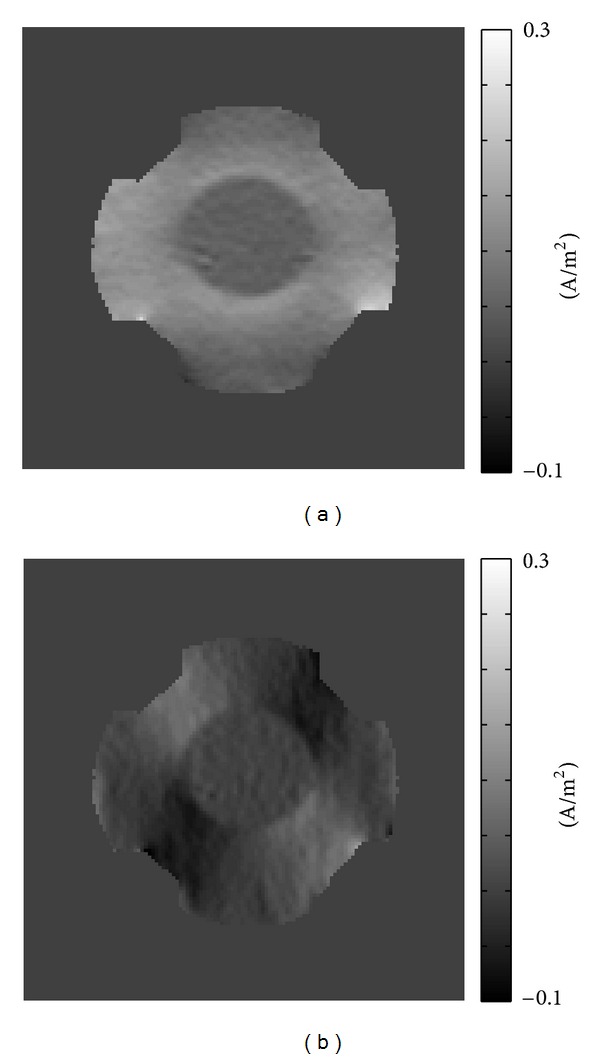
Recovered current density by solving (20) and (21). (a) Reconstructed *J*_*x*_^opt^ images, (b) *J*_*y*_^opt^ images by using *B*_*z*_^opt^.

We recovered the current density **J** ^opt^ by solving (20) and (21) using the optimized ${\tilde{\nabla }}^{2}B_{z}^{\text{opt}}=\sum_{\ell =1}^{N_{E}}\omega^{\ell }{\tilde{\nabla }}^{2}B_{z}^{\ell }$, where *ω* ^*ℓ*^ is the weighting factor by solving (17). The recovered *J*_*x*_^opt^ and *J*_*y*_^opt^ are displayed in Figure 7.

Table 2 shows the estimated noise level of the recovered ${\tilde{\nabla }}^{2}B_{z}^{\text{avrg}}$ and ${\tilde{\nabla }}^{2}B_{z}^{\text{opt}}$, by calculating (22). The estimated noise levels validate the proposed method because the inside and outside of the balloon in the phantom should be homogeneous.

## 4. Discussion

 Since the MREIT technique conventionally used the interleaved phase encoding acquisition scheme to measure the magnetic flux density by alternating two currents with positive and negative polarities, we could obtain the coil sensitivity information without additional scans by product of *ψ* ^*c*_*j*_+^ and *ψ* ^*c*_*j*_−^:

(24) Ψcj(x,y)∶=ψcj+(x,y)ψcj−(x,y)  =(ρcj(x,y))2e2iδcj(x,y), j=1,…,NC,

where *δ*_*c*_*i*__ is the *j*th coil sensitivity and *N*_*C*_ is the number of coils. For a fast MRI, using the * a priori* spatial information from the multiple receiver coils, the sensitivity encoding (SENSE) technique enables one to reduce the number of Fourier encoding steps while preserving the spatial resolution [32]. For a temporal variation of the internal conductivity, if we estimate the reference coil sensitivity using (24), which is independent of the injected current, the SENSE technique can be applicable to the proposed method to visualize the internal current density combining the multi-echo train.

In this paper, we directly measure ${\tilde{\nabla }}^{2}B_{z}$, which is sufficient to reconstruct the internal current density using the injected current information. The proposed method to measure ${\tilde{\nabla }}^{2}B_{z}$ in (14) can avoid a tedious unwrapping procedure. The proposed method may exhibit potential to be applied for conventional phase imaging techniques.

The optimal combination of multiple echoes by determining optimal weighting factor in (17) effectively reduces the noise level of measured ${\tilde{\nabla }}^{2}B_{z}$. Since the decay rate of magnitude and the width of injected current can be determined pixel by pixel, we can determine a pixel-wise noise level of the optimized ${\tilde{\nabla }}^{2}B_{z}$ data. Since most algorithms for the MREIT technique visualize the internal conductivity and/or current density in an entire imaging region due to the relationships between the external injection current and the internal measured magnetic flux density data, the estimated noise level of *B*_*z*_ can be used to determine the denoising level of the measured data in defective regions.

To optimize the multiple echoes, we consider only the uniformly distributed random noise effect, but unavoidable spike or different nonuniform noise may deteriorate a combined measured data. Thus it is important to develop a method to discard the non-uniform noises in the optimizing process in order to enhance the quality of *B*_*z*_.

Our future studies will focus on reducing the imaging time with a feasible noise level to produce conductivity images for the application of functional MREIT imaging to animal brains in order to visualize the rapidly changing conductivity associated with neural activation.

## 5. Conclusion

 We have visualized the internal current density using a fast ICNE-multi-echo MR pulse sequence based on a gradient echo by two measurements in the interleaved acquisition. The interleaved acquisition method in MREIT is a conventional method to suppress the background field inhomogeneity phase artifact and to increase the SNR of *B*_*z*_ by doubling the accumulated phase signal. We used the multi-echo pulse sequence, which acquires multiple sampling points within each repetition time. The proposed method directly measures the Laplacian of *B*_*z*_ from the measured *k*-space data, which can avoid a tedious unwrapping procedure and include a denoising effect by removing a part of the measured noise. We determined an optimal combination of the magnetic flux densities from the multi-echo in order to reduce the noise level. Using the optimization of ∇^2^ *B*_*z*_, the proposed method visualized the internal current density using the relationships between the induced internal current and the measured ∇^2^ *B*_*z*_ data, while suppressing the background field inhomogeneity. A real phantom experiment with a saline solution including a balloon was carried out to verify that the proposed method can be feasibly applied in real experiments. The total scan time in the phantom experiment was less than 13 seconds to visualize the current density with a 128 × 128 spacial matrix size.

## Figures and Tables

**Table 1:** Noise level estimation of the measured ${\tilde{\nabla }}^{2}B_{z}^{\ell }$, *ℓ* = 1,…, 9, by calculating (22).

	1st	2nd	3rd	4th	5th	6th	7th	8th	9th
Err_2_	129.1	142.1	115.0	110.4	203.1	161.7	195.6	183.5	166.6

**Table 2:** Noise level estimation of the recovered ${\tilde{\nabla }}^{2}B_{z}^{\text{avrg}}$ and ${\tilde{\nabla }}^{2}B_{z}^{\text{opt}}$, by calculating (22).

	${\tilde{\nabla }}^{2}B_{z}^{\text{avrg}}=\left(1/N_{E}\right)\sum_{\ell =1}^{N_{E}}{\tilde{\nabla }}^{2}B_{z}^{\ell }$	${\tilde{\nabla }}^{2}B_{z}^{\text{opt}}=\sum_{\ell =1}^{N_{E}}\omega^{\ell }{\tilde{\nabla }}^{2}B_{z}^{\ell }$
Err_2_	145.1	41.5
